# Effects of a multidisciplinary team-led school-based human papillomavirus vaccination health-promotion programme on improving vaccine acceptance and uptake among female adolescents: A cluster randomized controlled trial

**DOI:** 10.1097/MD.0000000000022072

**Published:** 2020-09-11

**Authors:** Janita Pak Chun Chau, Suzanne Hoi Shan Lo, Kai Chow Choi, Vivian Wing Yan Lee, Grace Chung Yan Lui, Kam Ming Chan, Alexander Yuk Lun Lau

**Affiliations:** a The Nethersole School of Nursing, Faculty of Medicine, The Chinese University of Hong Kong; b Centre for Learning Enhancement And Research, The Chinese University of Hong Kong; c Department of Medicine and Therapeutics, Faculty of Medicine, The Chinese University of Hong Kong; d Department of Paediatrics and Adolescent Medicine, United Christian Hospital, Hospital Authority, Hong Kong.

**Keywords:** adolescent, papillomavirus vaccines, randomized controlled trial, school health services, vaccination

## Abstract

**Introduction:**

Evidence has consistently shown the high efficacy of human papillomavirus (HPV) vaccines in preventing cervical cancers. However, the HPV vaccine uptake rate in Hong Kong is very low. We will develop and evaluate an innovative, theory-based multidisciplinary team-led school-based HPV vaccination health-promotion program (MDL-SHPVP), engaging female adolescents, parents/guardians, and secondary school personnel in multicomponent educational strategies and interactive discussions.

**Methods and analysis:**

A cluster randomized controlled trial is proposed. We will recruit 2520 female adolescents and their parents/guardians from 18 secondary day schools. The MDL-SHPVP is underpinned by the Health Belief Model and Precaution Adoption Process Model. Multicomponent interventions will be offered, including education sessions with small group dialogues with a registered nurse and trained healthcare and lay volunteers, and educational computer games. A team of volunteers will be established to raise HPV, cervical cancer, and HPV vaccine awareness. Outcomes include adolescents’ uptake of the HPV vaccine, adolescents’ intention to receive HPV vaccination, vaccine acceptance among parents/guardians, and parents’/guardians’ and adolescents’ HPV knowledge, attitudes, and beliefs. Data will be collected at baseline, 1 month, and 1 year after intervention. The generalized estimating equations analysis will be used for comparing the outcomes between the 2 groups.

**Ethics and dissemination:**

Ethical approval was obtained from the Joint Chinese University of Hong Kong-New Territories East Cluster Clinical Research Ethics Committee (Ref. no.: 2019.055). We will disseminate the study findings via peer-reviewed publications and presentations at relevant events and international and local conferences.

**Trial registration number:**

ClinicalTrials.gov NCT04438291

## 1 Introduction

Human papillomavirus (HPV) is the most common sexually transmitted infection, causing genital warts and anogenital cancers. It also causes cervical cancer, the fourth most common cancer in women, with 570,000 new cases reported worldwide in 2018.^[1,2]^ In Hong Kong (HK), cervical cancer has been the seventh most common cancer in women since 2015, and was the ninth leading cause of female cancer deaths in 2016.^[3]^ However, 3 types of HPV vaccines are currently available.^[1]^ Epidemiological studies consistently show the high safety and efficacy of HPV vaccines in preventing 90% of cervical cancers, 82% of high-grade anogenital pre-cancerous lesions, and 90% of genital warts. The protection lasts up to 10 years.^[4–6]^ Since 2007, it has been recommended that all women between 9 and 26 years of age receive HPV vaccines with a regimen of 2 to 3 doses, preferably at an earlier age before becoming sexually active. The highest level of immunogenicity and protection is resulted before exposure to the virus.^[1]^ Evidence has also suggested the potential development of herd immunity in the non-vaccinated population, including men.^[7]^ Nevertheless, a recent global estimate of HPV vaccination coverage among women between 9 and 26 years of age was only 39.7%.^[8]^

Many countries, such as Canada and Australia, have provided government-funded, free national HPV vaccination programs at schools since 2007 for girls aged 12 to 13 years.^[9,10]^ In HK, HPV vaccines are available from local registered physicians, costing HK$1200 to HK$1800 per dose. Teenage girls from low-income families can also join the 3-year Cervical Cancer Vaccination Pilot Scheme, launched in 2016 and supported by the Community Care Fund.^[11]^ Regardless, a systematic review reported an extremely low vaccine uptake rate among HK adolescents (2.4%–9.1%), similar to that in Western countries.^[12]^ Consequently, a new vaccination scheme is offering free HPV vaccination to Primary Five and Primary Six schoolgirls from the academic year, 2018 to 2019.^[13]^ However, current secondary school female adolescents are not covered. Western experiences show that although government-funded HPV vaccination increase the HPV vaccine uptake rate, there exist other barriers hindering adolescents’ or parents/guardians’ decision to initiate and complete HPV vaccination.^[14,15]^ Effective interventions to address these barriers are warranted.

Adolescents’ and parents’ knowledge and attitudes towards HPV and HPV vaccine are crucial in their decision to vaccinate.^[16,17]^ One systematic review of 25 studies found that vaccine uptake among female adolescents was associated with vaccine-related knowledge, attitudes, information provided by healthcare providers, health insurance coverage, age, and childhood vaccination.^[18]^ Another systematic review of 28 qualitative studies and 44 surveys reported the consistent finding that adolescents and parents struggled with their decision due to limited information, declining vaccination for concerns over vaccine safety and low perception of risk of HPV infection.^[19]^ A cross-sectional survey of 1416 female adolescents from 8 secondary schools in HK reported a significant knowledge gap regarding cervical cancer prevention. The 3 most significant factors influencing vaccination include the perception of cancer as a terrifying disease, the provision of more information on cancer prevention from schools and comments from vaccinated relatives and friends. The cost of vaccination and socio-economic background were not significant.^[20]^ Another review also highlighted that perceived beliefs of peers and significant others are associated with HPV vaccination.^[21]^

Previous studies have examined various educational interventions enhancing HPV vaccination uptake. A systematic review of 33 studies found that various educational interventions directed at adolescents or young adults, or their parents increased knowledge, positive intentions, and attitudes. However, they did not lead to an increased rate of HPV vaccine uptake as the rise in the rate of intention was extinguished after 1 month. The review further found a lack of educational interventions explicitly underpinned by theoretical frameworks.^[22]^ A cluster randomized controlled trial (RCT) of 832 students from 18 upper secondary schools in Sweden revealed that a school-based educational intervention underpinned by the Health Belief Model (HBM) resulted in a significantly greater proportion of students in the intervention group deciding to receive HPV vaccination. Significant improvements in condom use and the HBM total susceptibility and severity scores were also seen in the intervention group relative to the control group.^[23]^ Another study of 383 undergraduate students adopted the Precaution Adoption Process Model (PAPM) and found that most unvaccinated students were in the early stages of decision-making regarding vaccination. They perceived low susceptibility of themselves to HPV, but had basic knowledge about HPV. The study suggested that it would be helpful to provide prompts from healthcare providers and education on susceptibility according to students’ stages of decision-making to enhance vaccination rates.^[24]^

Additionally, 2 HK studies reported the effects of educational interventions on HPV uptake and intention. A pre-post study of 953 female adolescents found an 11.3% increase in the number of participants intending to receive HPV vaccination after school-based cervical cancer education underpinned by theories of persuasion. Participants also showed greater knowledge in and a more positive attitude towards HPV vaccination.^[25]^ Another non-profit-funded school-based program using HBM provided free HPV vaccination and educational interventions to 1229 female adolescents (9–14 years) from low-income families. Promising results indicated overall vaccine uptake rates of 81.4% and 80.8% for the first and second doses, respectively. The results showed an association between parents who did not want their daughters vaccinated and inadequate knowledge regarding the vaccine.^[17]^

It is evident that providing sufficient knowledge and conveying positive attitudes towards HPV vaccination among adolescents and their parents/guardians are very important in increasing the HPV vaccine uptake rate. Educational interventions involve a complex design and evaluation driven by theoretical premises crucial to better understanding causal intervention mechanisms and changes in outcomes.^[26]^ The only HK study in which an intervention was developed on the basis of HBM was a one-group pre/post-test study.^[17]^ Further evaluation using a RCT is needed to demonstrate intervention effects on actual vaccine uptake instead of simply on intention to be vaccinated. Furthermore, although previous studies targeted adolescents and/or parents, it would be more comprehensive to include school personnel to better equip them to provide health information to students. Partnerships are needed between schools, students, and healthcare professionals. With the government's introduction of a free HPV vaccination program for primary school girls, the provision of educational interventions for them is obviously necessary. However, it is important to provide the same interventions to secondary school female adolescents requiring catch-up doses. Booster doses of educational interventions are also needed in view of the decreasing uptake rate of the second and third HPV vaccines observed in previous studies. Studies have supported the feasibility of conveying information via volunteers. Hence, more strengthening strategies, including examining the feasibility of training a team of healthcare and lay volunteers for community capacity building to sustain vaccination uptake, are needed.

## 2 Methods

### 2.1 Study design

A cluster RCT is proposed.

### 2.2 Objectives

The study aims to examine the effects of a multidisciplinary team-led school-based HPV vaccination health-promotion program (MDL-SHPVP) on female adolescents and their parents or guardians. The primary outcome is female adolescents’ uptake of the HPV vaccine 1 year after intervention. The secondary outcomes are female adolescents’ intention to receive HPV vaccination, vaccine acceptance among their parents/guardians, and parents/guardians’ and female adolescents’ HPV knowledge, attitudes, and beliefs (from baseline to 1 month after intervention).

### 2.3 Participants

We will recruit 2520 adolescents and their parents/guardians from 18 secondary day schools (out of 471 secondary schools). Six schools will be randomly selected from each of the 3 regions of HK (HK Island, Kowloon, and New Territories) using a sample frame compiled from the school lists by district available at the Education Bureau. Adolescents admitted to the trial will be 14- to 17-year-old secondary school female students (seventh to eleventh grade) who have not received an HPV vaccine. Exclusion criteria will include: history of severe life-threatening allergies to a previous dose of HPV vaccine or vaccine component; history of immediate hypersensitivity to yeast; moderate or severe acute illness^[27]^; and participation in previous trials related to HPV vaccination. Only 1 daughter from each family will be selected. Parents/guardians admitted to the trial must have a daughter meeting the above criteria; live with their daughter and spend the most time caring for her; be able to communicate in Chinese; and be willing to attend education sessions. Parents/guardians will be excluded if they currently suffer from psychiatric illness.

### 2.4 Sample size estimation

Sample size is estimated based on the primary outcome of the HPV vaccine uptake rate. Several studies have revealed the low uptake rate of HPV vaccine (<10%) in HK.^[20,28,29]^ We anticipate that our proposed MDL-SHPVP can double the uptake rate, and have therefore set this target to determine its effectiveness a priori. We used the power analysis software PASS 14 (NCSS, Kaysville, UT) to estimate that a sample size of 266 participants in the intervention and control groups will be required to achieve 80% power at a 2-sided 5% level of significance to detect a net difference of at least 10% in the uptake rate between the 2 groups, assuming that the rate is 10% for the control group. To allow for an attrition rate of up to 20%, a total of 666 participants with 333 in each group are needed. Furthermore, to account for clustering design, a variance inflation factor, namely the design effect, must be imposed.^[30]^ The design effect is given by 1 + (*m*–1)∗ICC, where *m* is the average cluster size and ICC is the intracluster correlation coefficient of the underlying outcome. A synthesis study revealed that the ICC tends to be small in primary care research, with a median of 0.005 and an inter-quartile range of 0.000 to 0.021.^[31]^ As we do not have empirical data to estimate the ICC for the outcome, we account for an ICC of up to 0.02. By this standard, a total of 18 schools with at least 140 female adolescents from each school will be required.

### 2.5 Cluster randomization

Randomization will be performed at the school level (cluster) rather than the individual participant level to avoid contamination between the intervention and control groups. Schools will be randomized to either the intervention or control group in a 1:1 ratio. All eligible adolescents recruited from the same school will be allocated to either the intervention or control group. Group allocation will be concealed from outcome assessors and the investigators, and performed according to a random sequence generated by an independent statistician using computer-based randomization.

### 2.6 Intervention schools

Multicomponent interventions will be offered, including 5 identical 2-hour education sessions to adolescents and parents/guardians in each school (28 adolescents and 28 parents/guardians in each session). The project nurse will assume the role of “HPV immunization advisor” and pair with healthcare and lay volunteers to facilitate small-group discussions, encouraging engagement, and dialogue. The topics introduced in these identical sessions will include the mechanism of spread of HPV; diseases or conditions caused by HPV; HPV prevention; incidence of cervical cancer, its symptoms and complications, high-risk groups, and prevention; efficacy and safety of HPV vaccines; recommendations and duration of protection by HPV vaccine; concept of herd immunity; benefit–risk ratios; common misconceptions and concerns; and concerns about costs, safety, and side effects of vaccines. The education sessions will be offered after school hours, including evenings and weekends to fit the schedules of working parents/guardians. Online educational computer games will also be introduced to adolescents. These games will encourage adolescents to learn about the concept of vaccination as an effective preventive measure against HPV, resolve common misconceptions, and provide justifications for HPV vaccination.

To assure program sustainability, a 2-hour education session will be offered to school personnel, including teachers, school nurses, social workers, and administrative staff. All teaching and learning materials will be rigorously reviewed by a multidisciplinary team of nurse consultants, nurse academics, physicians, pharmacists, and infectious disease specialists to ensure content validity. A pilot study of 3 health education sessions will be conducted with 9 participants (including adolescents, parents/guardians, and teachers). Participants will be asked to comment on the acceptability and feasibility of the education sessions and pre-test the instruments to identify issues related to data collection. Five 3-hour training sessions lead by 4 experts in pediatric nursing, public health, pharmacology, and infectious diseases will be provided to build upon project nurses’ skills and experience in delivering the intervention. Topics will include therapeutic communication skills with adolescents, parents/guardians and teachers; vaccine pharmacology; HPV infection and prevention; empowerment of parents/guardians and adolescents to make healthy decisions; and the innovative application of computer games in healthcare decisions.

Regarding capacity building, we will establish a core team of volunteers, including 30 healthcare volunteers (physicians, registered nurses, pharmacists, and social workers) and 120 lay volunteers (university students), to assist in the education sessions (at least 1 healthcare and 3 lay volunteers per session). All volunteers will attend a 2-day training session conducted by the project team on health education and promotion theories; communication skills; knowledge about cervical cancer; HPV; and HPV vaccination. Ten video-based scenarios about strategies to engage adolescents and their parents/guardians in open dialogue (including good and poor practices) will be developed. Discussions, role-playing sessions, and adolescents and parents/guardians simulation practice will be conducted. A training manual will be developed to summarize the training contents. All volunteers must pass a quiz and perform satisfactorily in an encounter with 2 pairs of simulated participants of an adolescent and their parent/guardian. A certificate will be issued after completion of training.

### 2.7 Control schools

Parents/guardians and adolescents from control schools will receive a 30-minute educational video on HPV, cervical cancer, and the HPV vaccine designed to enhance HPV vaccine acceptance. A video is provided to avoid exclusion of any parents/guardians who may be illiterate.

### 2.8 Outcome measures

Outcomes will be determined 1 month after the intervention (i.e., the education sessions) (T1) and 1 year after the intervention (T2) and will be collected by a research assistant unaware of the group allocation. Adolescents and their parents/guardians will be asked to report their immunization status during the 1-year follow-up (T2).

#### 2.8.1 Immunization status and intention

HPV vaccine uptake (when and where) as determined by the proportion of female adolescents vaccinated; and adolescents’ intention to vaccinate on a 10-point Likert scale (1 = definitely not, 10 = definitely)^[32]^ and reasons for no or low intention (a score <5) will be sought.

#### 2.8.2 Parents/guardians’ acceptance of HPV vaccination

The 6 PAPM stages proposed by Shapiro et al^[33]^ will be used to identify the stage of parents/guardians’ HPV vaccine decision-making at baseline (T0) and T1. The 6 stages include unaware of the vaccine; unengaged in the decision to vaccinate their daughter; undecided; decided not to act; decided to act; and already acted (vaccinated their daughter). The PAPM is a stage-based theoretical model, aiding better understanding of the vaccine acceptance process among respondents.^[34]^

#### 2.8.3 Parents/guardians’ and adolescents’ attitudes and beliefs regarding HPV vaccination

The 18-item Carolina HPV Immunization Attitudes and Beliefs Scale (CHIAS)^[34]^ based on HBM constructs will be used to measure parents/guardians’ attitudes and beliefs about the HPV vaccine. Participants will rate each item on a 4-point scale, with higher values denoting stronger agreement. An example of a statement in this scale is “The HPV vaccine might cause lasting health problems.” The CHIAS examined HPV vaccine decision-making and was validated in a group of 783 parents who had not vaccinated their daughters. The 4 CHIAS factors include “perceived vaccination harms,” “perceived barriers,” “perceived vaccine effectiveness,” and “uncertainty about the vaccine.” All factors have acceptable internal consistencies, with Cronbach alpha (*α*) values ranging from 0.61 to 0.69.^[34]^

The 17-item modified CHIAS^[35]^ will be used to measure adolescents’ attitudes and beliefs about the HPV vaccine. Participants will rate each item on an 11-point scale, with higher values denoting lower endorsement of HPV vaccination. The scale was validated in 139 college-aged women and yielded a factor structure similar to studies with parents. Three of the factors (barriers, harms, and effectiveness) show good internal consistency, with Cronbach *α* from 0.74 to 0.90, while the other 2 factors (uncertainty and risk denial) have Cronbach *α* of 0.43 to 0.49.^[35]^

#### 2.8.4 Adolescents’ and parents’/guardians’ knowledge about HPV and HPV vaccine

A 23-item HPV knowledge scale (GK23) and a 9-item vaccination knowledge scale (VK9)^[36]^ will be used to measure adolescents’ and parents/guardians’ knowledge about HPV and the HPV vaccine. Participants will be asked to respond “true,” “false,” or “don’t know” to 32 items. Both scales have been validated and used in studies in which parents’ knowledge about HPV and the HPV vaccine was examined.^[36]^ The GK23 has high internal consistency, with a Cronbach *α* of 0.89. The Cronbach *α* of VK9 is 0.68.

All instruments will be translated into Chinese, blind back-translated, reviewed, and modified by independent bilingual persons. The translated instruments will be validated with a group of 100 female secondary school students and 100 parents. An online survey of these instruments will be conducted.

#### 2.8.5 Socio-demographic and vaccination information

Information collected will comprise: parents/guardians’ age, sex, educational level, occupation, income, and current and past medical history; number of children in the family; adolescents’ age and immunization history; receipt of influenza and HPV vaccine, and educational talks on HPV vaccination; HPV immunization of family members; and whether vaccinations have been discussed with healthcare professionals.

### 2.9 Data collection methods

Adolescents in both groups will be asked to complete the online surveys at 3 time points: T0, after receiving parental and adolescent consent; T1, 1 month after the program (T0 and T1 will assess both adolescents’ intention to receive HPV vaccine and HPV knowledge, attitudes, and beliefs); and T2, 1 year after the program, at which time we will collect information about adolescents’ uptake of the HPV vaccine. Telephone or online surveys will be administered to parents/guardians in both groups at the same time points: at T0 and T1, to assess vaccine acceptance among parents/guardians and their HPV knowledge, attitudes and beliefs; and at T2, to collect information about the uptake of HPV vaccine among their daughters. In addition, satisfaction data from adolescents, parents/guardians, and teachers of both groups will be collected at T1 (via online survey for adolescents, and telephone or online survey for parents/guardians and teachers). Feedback from project nurse and volunteers will be collected at T1 (via telephone interviews). Research assistant, who will collect the study outcome data (except the satisfaction data for both groups collected by another research assistant) and perform data entry and analysis will remain blinded to the treatment allocation.

### 2.10 Data analysis

Data will be summarized and presented using appropriate descriptive statistics. The normality of continuous variables will be assessed using skewness and kurtosis statistics and a normal probability plot. Suitable transformations will be made on skewed variables before subjecting them to inferential analysis, if necessary. The homogeneity of participants’ characteristics between the 2 groups will be evaluated by independent *t*, chi-squared or Fisher exact tests, as appropriate. The generalized estimating equations (GEE) analysis with small-sample correction for sandwich variance estimate, which can be used to account for the small number of clusters in cluster randomized trial,^[37]^ will be used for comparing the primary and secondary outcomes between the 2 groups. The GEE analysis will be performed with the procedure GLIMMIX implemented in SAS release 9.4 (SAS Institute Inc., Cary, NC). All statistical tests involved are 2-sided, with the level of significance set at 0.05.

### 2.11 Patient and public involvement

User engagement will be promoted via user-centered design methods.^[38]^ Twenty-six potential users and key stakeholders, including 8 parents, 8 adolescents, 4 secondary school teachers, 2 school principals, 2 school nurses, and 2 social workers, will be engaged in the design of the MDL-SHPVP. Discovery interviews will be conducted to obtain a range of perspectives and insights and examine their perceived information needs, views of HPV vaccination, and program expectations. They will also be asked to review the deliverables, and a cycle of feedback loops will be initiated between them and the project team.

### 2.12 Reporting guidelines

SPIRIT reporting guidelines were adhered in this protocol.^[39]^

## 3 Ethics and dissemination

Ethical approval was obtained from the Joint Chinese University of Hong Kong-New Territories East Cluster Clinical Research Ethics Committee (Ref. no.: 2019.055) on March 13, 2019. We will protect participants’ rights and safety by adhering to local laws, the Hong Kong Personal Data (Privacy) Ordinance, the Declaration of Helsinki, institutional policies, and the ICH-GCP. Eligible schools and participants will receive an explanation of the project, potential risks or benefits, and their right to voluntary participation, withdrawal from the study at any time without negative consequences, and confidentiality. Informed consent will be obtained. Consent forms with the option of participation for parents/guardians will be sent home with adolescents. All information collected will be kept strictly confidential. Study findings will be disseminated via peer-reviewed publications and presentations at relevant events and conferences.

## 4 Study timeline

The study will be conducted over a period of 30 months. Recruitment of participants is estimated to begin in December 2020 and will continue over 12 months. It will be followed with baseline measurement, intervention delivery, follow-up measurement, and results analysis, writing, and dissemination.

## Author contributions

Janita Pak Chun Chau and Suzanne Hoi Shan Lo conceived and designed the study. Janita Pak Chun Chau, Suzanne Hoi Shan Lo, Kai Chow Choi, Vivian Wing Yan Lee, Grace Chung Yan Lui, Kam Ming Chan, and Alexander Yuk Lun Lau prepared the protocol. All authors have read and approved the manuscript.

## References

[1] World Health Organization. Cervical cancer. Available at: http://www.who.int/cancer/prevention/diagnosis-screening/cervical-cancer/en/ [accessed June 3, 2020].

[2] Oh JK Weiderpass E Infection and cancer: global distribution and burden of diseases. Ann Glob Health 2014;80:384 92. 25512154 10.1016/j.aogh.2014.09.013

[3] Centre for Health Protection. Cervical cancer. Available at: https://www.chp.gov.hk/en/healthtopics/content/25/56.html [accessed June 3, 2020].

[4] Harper DM DeMars LR HPV vaccines - a review of the first decade. Gynecol Oncol 2017;146:196 204. 28442134 10.1016/j.ygyno.2017.04.004

[5] Lee LY Garland SM Human papillomavirus vaccination: the population impact. F1000Res 2017;6:866 77. 28663791 10.12688/f1000research.10691.1 PMC5473416

[6] Lehtinen M Lagheden C Luostarinen T Ten-year follow-up of human papillomavirus vaccine efficacy against the most stringent cervical neoplasia end-point-registry-based follow-up of three cohorts from randomized trials. BMJ Open 2017;7:e015867 75. 10.1136/bmjopen-2017-015867 PMC5629648 28821519

[7] Brisson M Bénard É Drolet M Population-level impact, herd immunity, and elimination after human papillomavirus vaccination: a systematic review and meta-analysis of predictions from transmission-dynamic models. Lancet Public Health 2016;1:e8 17. 29253379 10.1016/S2468-2667(16)30001-9 PMC6727207

[8] Bruni L Diaz M Barrionuevo-Rosas L Global estimates of human papillomavirus vaccination coverage by region and income level: a pooled analysis. Lancet Glob Health 2016;4:e453 63. 27340003 10.1016/S2214-109X(16)30099-7

[9] Shapiro GK Guichon J Kelaher M Canadian school-based HPV vaccine programs and policy considerations. Vaccine 2017;35:5700 7. 28893472 10.1016/j.vaccine.2017.07.079

[10] Australian Government Department of Health. National Immunisation Program Schedule. Available at: https://www.health.gov.au/health-topics/immunisation/immunisation-throughout-life/national-immunisation-program-schedule%E3%2A [accessed June 3, 2020].

[11] The Nethersole School of Nursing, Faculty of Medicine, The Chinese University of Hong Kong. Human papillomavirus (HPV) booklet. Available at: http://www.nur.cuhk.edu.hk/wp-content/uploads/2019/06/CUHK-HPV-booklet_v8.pdf [accessed June 3, 2020].

[12] Loke AY Kwan ML Wong YT The uptake of human papillomavirus vaccination and its associated factors among adolescents: a systematic review. J Prim Care Community Health 2017;8:349 62. 29161946 10.1177/2150131917742299 PMC5932744

[13] The Hong Kong Special Administrative Region of the People's Republic of China. The Chief Executive's 2018 Policy Address: VI. Improving People's Livelihood. Available at: https://www.policyaddress.gov.hk/2018/eng/policy_ch06.html [accessed June 3, 2020].

[14] Rose SB Lawton BA Lanumata T HPV/cervical cancer vaccination: parental preferences on age, place and information needs. J Prim Health Care 2010;2:190 8. 21069114

[15] Vercruysse J Chigurupati NL Fung L Parents’ and providers’ attitudes toward school-located provision and school-entry requirements for HPV vaccines. Hum Vaccin Immunother 2016;12:1606 14. 26934421 10.1080/21645515.2016.1140289 PMC4964627

[16] Bartlett JA Peterson JA The uptake of human papillomavirus (HPV) vaccine among adolescent females in the United States: a review of the literature. J Sch Nurs 2011;27:434 46. 21750234 10.1177/1059840511415861

[17] Yuen WWY Lee A Chan PKS Uptake of human papillomavirus (HPV) vaccination in Hong Kong: facilitators and barriers among adolescent girls and their parents. PLoS One 2018;13:e0194159 74. 29543839 10.1371/journal.pone.0194159 PMC5854366

[18] Kessels SJ Marshall HS Watson M Factors associated with HPV vaccine uptake in teenage girls: a systematic review. Vaccine 2012;30:3546 56. 22480928 10.1016/j.vaccine.2012.03.063

[19] Hendry M Lewis R Clements A HPV? Never heard of it!”: a systematic review of girls’ and parents’ information needs, views and preferences about human papillomavirus vaccination. Vaccine 2013;31:5152 67. 24029117 10.1016/j.vaccine.2013.08.091

[20] Lee A Ho M Cheung CKM Factors influencing adolescent girls’ decision in initiation for human papillomavirus vaccination: a cross-sectional study in Hong Kong. BMC Public Health 2014;14:925 35. 25195604 10.1186/1471-2458-14-925 PMC4176578

[21] Gamble HL Klosky JL Parra GR Factors influencing familial decision-making regarding human papillomavirus vaccination. J Pediatr Psychol 2010;35:704 15. 19966315 10.1093/jpepsy/jsp108 PMC2915623

[22] Fu LY Bonhomme LA Cooper SC Educational interventions to increase HPV vaccination acceptance: a systematic review. Vaccine 2014;32:1901 20. 24530401 10.1016/j.vaccine.2014.01.091 PMC4285433

[23] Grandahl M Rosenblad A Stenhammar C School-based intervention for the prevention of HPV among adolescents: a cluster randomised controlled study. BMJ Open 2016;6:e009875 86. 10.1136/bmjopen-2015-009875 PMC4735203 26817639

[24] Barnard M George P Perryman ML Human papillomavirus (HPV) vaccine knowledge, attitudes, and uptake in college students: implications from the Precaution Adoption Process Model. PLoS One 2017;12:e0182266 75. 28786994 10.1371/journal.pone.0182266 PMC5546631

[25] Kwan TTC Tam KF Lee PWH The effect of school-based cervical cancer education on perceptions towards human papillomavirus vaccination among Hong Kong Chinese adolescent girls. Patient Educ Couns 2011;84:118 22. 20650592 10.1016/j.pec.2010.06.018

[26] Craig P Dieppe P Macintyre S Medical Research Council Guidance. Developing and evaluating complex interventions: the new Medical Research Council guidance. Int J Nurs Stud 2013;50:587 92. 23159157 10.1016/j.ijnurstu.2012.09.010

[27] Centers for Disease Control and Prevention. HPV Vaccine Recommendations. Available at: https://www.cdc.gov/vaccines/vpd/hpv/hcp/recommendations.html [accessed June 3, 2020].

[28] Choi HC Leung GM Woo PP Acceptability and uptake of female adolescent HPV vaccination in Hong Kong: a survey of mothers and adolescents. Vaccine 2013;32:78 84. 24188759 10.1016/j.vaccine.2013.10.068

[29] Li SL Lau YL Lam TH HPV vaccination in Hong Kong: uptake and reasons for non-vaccination amongst Chinese adolescent girls. Vaccine 2013;31:5785 8. 24148571 10.1016/j.vaccine.2013.10.027

[30] Donner A Klar N Cluster randomization trials in epidemiology: theory and application. J Stat Plan Inference 1994;42:37 56.

[31] Adams G Gulliford MC Ukoumunne OC Patterns of intra-cluster correlation from primary care research to inform study design and analysis. J Clin Epidemiol 2004;57:785 94. 15485730 10.1016/j.jclinepi.2003.12.013

[32] Hofman R Schiffers PA Richardus JH Increasing girls’ knowledge about human papillomavirus vaccination with a pre-test and a national leaflet: a quasi-experimental study. BMC Public Health 2013;13:611 8. 23802703 10.1186/1471-2458-13-611 PMC3698036

[33] Shapiro GK Perez S Naz A Investigating Canadian parents’ HPV vaccine knowledge, attitudes and behaviour: a study protocol for a longitudinal national online survey. BMJ Open 2017;7:e017814 22. 10.1136/bmjopen-2017-017814 PMC5652458 29025844

[34] McRee AL Brewer NT Reiter PL The Carolina HPV immunization attitudes and beliefs scale (CHIAS): scale development and associations with intentions to vaccinate. Sex Transm Dis 2010;37:234 9. 19940807 10.1097/OLQ.0b013e3181c37e15

[35] Dempsey AF Fuhrel-Forbis A Konrath S Use of the Carolina HPV Immunization Attitudes and Beliefs Scale (CHIAS) in young adult women. PLoS One 2014;9:e100193 9. 24945630 10.1371/journal.pone.0100193 PMC4063751

[36] Sherman SM Nailer E Attitudes towards and knowledge about human papillomavirus (HPV) and the HPV vaccination in parents of teenage boys in the UK. PLoS One 2018;13:e0195801 17. 29641563 10.1371/journal.pone.0195801 PMC5895045

[37] Leyrat C Morgan KE Leurent B Cluster randomized trials with a small number of clusters: which analyses should be used? Int J Epidemiol 2018;47:321 31. 29025158 10.1093/ije/dyx169

[38] Weberg D Davidson S Patient-centered care, evidence, and innovation. In: Davidson S, Weberg D, Porter-O’Grady T, et al., eds. Leadership for Evidence-based Innovation in Nursing and Health Professions. Burlington, MA: Jones & Bartlett Learning; 2017:111–141.

[39] Chan AW Tetzlaff JM Altman DG SPIRIT 2013 statement: defining standard protocol items for clinical trials. Ann Intern Med 2013;158:200 7. 23295957 10.7326/0003-4819-158-3-201302050-00583 PMC5114123

